# Factor-V Leiden G1691A and prothrombin G20210A polymorphisms in Sudanese women with preeclampsia, a case -control study

**DOI:** 10.1186/s12881-018-0737-z

**Published:** 2019-01-05

**Authors:** Nadir A. Ahmed, Ishag Adam, Salah Eldin G. Elzaki, Hiba A. Awooda, Hamdan Z. Hamdan

**Affiliations:** 1 Department of Biochemistry and Molecular Biology, Faculty of Medicine, Al-Neelain University, P.O. Box: 12702, Khartoum, Sudan; 2 Faculty of Medicine, University of Khartoum, Khartoum, Sudan; 3 Department of Epidemiology, Tropical Medicine Research Institute, Khartoum, Sudan

**Keywords:** Factor V Leiden1691G/A, prothrombin20210G/A, Polymorphism, Factor II, Factor V, Preeclampsia, Sudan

## Abstract

**Background:**

Preeclampsia can lead to adverse maternal and perinatal outcomes. There are few studies on the association of preeclampsia with thrombophilia in Africa including Sudan.

**Methods:**

A case –controls study was conducted at Saad Abualila Hospital in Khartoum, Sudan during the period of February through November 2017. The cases were women with preeclampsia and healthy pregnant women were the controls (180 women in each arm of the study). Genotyping for Factor-V Leiden 1691G/A and Prothrombin gene variation 20210G/A was done by polymerase chain reaction–restriction fragment length polymorphism (PCR–RFLP).

**Results:**

There was no significant difference in the age, parity, body mass index (BMI) and the other characteristics between the cases and the controls. Genotypes distribution of Factor V Leiden 1691G/A and prothrombin gene 20210G/A in controls was in accordance with the Hardy–Weinberg equilibrium (*P* > 0.05). The factor V Leiden-variation was present in 9.6% of the cases compared with 0.6% of the controls, *P* < 0.001 (OR = 18.60, 95% CI = 2.38–136.1). Only 4 patients with severe preeclampsia had homozygous variation A/A and it was not detected in the controls. Prothrombin G20210A variations not detected neither in the cases nor in the controls group.

**Conclusions:**

High prevalence of Factor V Leiden 1691G/A variation in preeclamptic patients compared to controls suggest an involvement of this variation in predisposing to preeclampsia in this setting.

## Background

Preeclampsia is defined as occurrence of hypertension during pregnancy and the presence of proteinuria after the 20th week of gestation in previously normotensive woman [1]. Preeclampsia is a multisystem disorder and one of the most common encountered serious pregnancy-related disease that affects around 3–8% of all human pregnancies [2, 3].

Although the exact etiology of preeclampsia is not fully understood, the main pathophysiology is the inadequate development of the early placenta “poor placentation”, reduced capacity of the utero-placental circulation which can lead to endothelial dysfunction and the initiation of preeclampsia [4, 5].

Preeclampsia has gene variations; however the precise patho-mechanism and genetic basis of preeclampsia remains unknown [6]. The association of Factor-V (rs6025) and Factor II (Prothrombin) (rs1799963) variations with preeclampsia and their possible role in the pathogenesis of preeclampsia have been previously investigated [7]. The results, however, are still inconclusive and contradictory. While some studies reported association between preeclampsia and Factor-V Leiden [8–10], others fail to find an association [11, 12]. Likewise, prothrombin G20210A variation were associated with sever preeclampsia in some studies [13, 14], others found no association [15, 16].

Preeclampsia/eclampsia is the major health problem and associated with high maternal and perinatal mortality in Sudan [17, 18]. There are few published data on Factor-V Leiden, Prothrombin G20210A variations and preeclampsia in Africa and there is no published data on Factor-V Leiden, Prothrombin G20210A variations and preeclampsia in Sudan [19, 20]. Therefore, the current study was conducted to assess Factor-V Leiden and Prothrombin variation in Sudanese women with preeclampsia.

## Methods

A case –controls study (180 women in each arm) was conducted at Saad Abualila Maternity Tertiary Hospital in Khartoum, Sudan during the period of February through November 2017.

Pregnant women with preeclampsia (blood pressure ≥ 140/90 mmHg on 2 occasions, at least 6 h apart, and proteinuria of ≥300 mg/24 h) were the cases. Preeclampsia was classified in mild and severe form. Severe preeclampsia was considered in the occurrence of one or more of the followings; blood pressure ≥ 160/110 mmHg, proteinuria of ≥5 g/24 h and HELLP syndrome “hypertension, proteinuria and presence of hemolysis, elevated liver enzymes and low platelet count” [1].

The controls were healthy pregnant women without hypertension or proteinuria or diabetes or any underlying disease such as thyroid disorders.

**Fig. 1:**
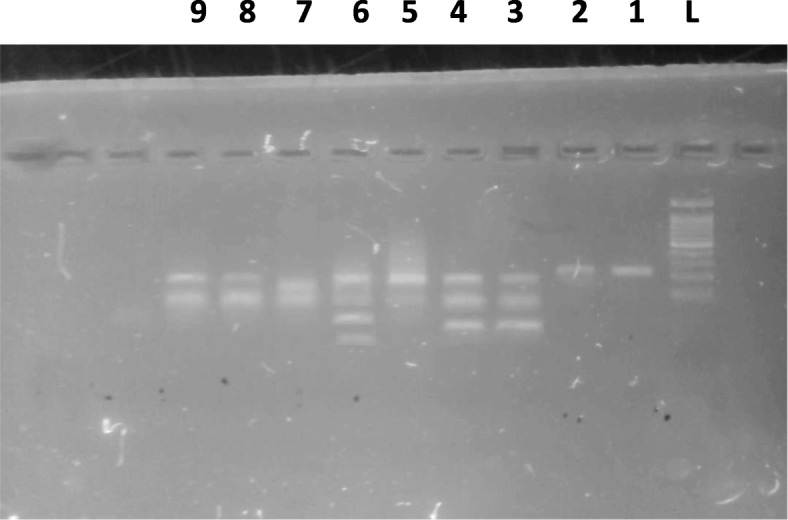
PCR-RFLP analysis for Factor-V Leiden G1691A polymorphism. Two fragments of 47 bp and 159 bp indicate mutant homozygous (A/A), three fragments of 36 bp, 47 bp and 123 bp for wild type homozygous (G/G) and four fragments of 36 bp, 47 bp, 123 bp and 159 bp indicate A/G genotype. Lane L show the 100 bp ladder; Lane 1 and 2 undigested PCR products. Lane 3 and 4 indicate wild type homozygous (G/G); 5, 7–9 homozygous mutant (A/A) and Lane 6 represents heterozygous (A/G) genotype

After signing an informed consent, clinical and obstetrics history (age, parity, and gestational age) were gathered using a questionnaire. The Body Mass Index (BMI) was computed from the measured weight and height as weight in kg/ square height in meter. Then 3 mL of whole blood collected in Ethylene diamine tetra acetic acid (EDTA) was used to extract DNA using salting out method [21]. The detection of Factor-V Leiden G1691A and Prothrombin G20210A variations was based on examination of the size of the polymerase chain reaction (PCR) products following DNA amplification of the target sequence of the Factor-V gene and factor II gene, respectively. Oligonucleotides used as primers were: 5’-CATACTACAGTGACGTGGAC-3′ and 5’-TGTTCTCTTGAAGGAAATGC-3′ for Factor-V Leiden; 5’-TCTAGAAACAGTTGCCTGGC-3′ and 5’-ATAGCACTGGGAGCATTGAAGC-3′ for the G20210A variation of the Prothrombin T gene. A reaction mixture consist of DNA, forward and revered primer and nuclease free water were added into PCR tubes using ready mix (Maxime PCR PreMix,i-Taq for 20 μl). The mixture was loaded into thermocycler according to the specific temperature profile. The PCR was performed in 35 cycles consisting of denaturation at 94 °C for 30 s, annealing at 63 °C for 30 s, extension at 72 °C for 30 s, and final extension at 72 °C for 5 min. 1.5% agarose (iNtRON, Biotechnology) was prepared from 1x TBE and 5 μl PCR products were loaded, run on the gel for 30 mins and visualized on UV transllimantor. The resulting PCR product for Factor-V 206-bp digested with 10 μl of DNA restriction enzyme *MnI*1 (New England Biolabs®Inc.), and resulted in 3 fragments their length are 36, 47 and 123 bp for wild allele G/G, 2 fragments for mutant allele (47 and 159 bp) A/A and heterozygote allele resulted in four fragments (36,47,123 and 159 bp) A/G, Fig. 1. While, Prothrombin PCR product 345 bp digested with 20 U of *Hind-III* (New England Biolabs®Inc.), and resulted in undigested PCR product for wild type G/G. Two fragments for mutant allele A/A (322 and 23 bp) and 3 fragments for heterozygote allele A/G (322, 23 and 345 bp). Genotyping for factor V and prothrombin is further confirmed by DNA sequencing for the PCR product by using the same primers pairs and it is done in Macrogen Inc., South Korea.

The sample size was calculated as for case -control study 1: 1ratio and the difference in the proportions of variation between the cases and controls. It was assumed that Factor-V Leiden variation would be in 7.0% of the pregnant women with preeclampsia and 1% in pregnant women with no complication. This rate was assumed from our previous report on Factor-V Leiden variation among pregnant Sudanese women with deep venous thrombosis [22]. This would give the study at least 80% power and the difference of 0.05 at *α* level [23].

### Statistics

Data were entered in computer sing SPSS for Windows for data analyses. The clinical data were compared between women with preeclampsia and controls by *t-test* and *chi-square* test for continuous and categorized data, respectively. Hardy–Weinberg equilibrium (HWE) was conducted by comparing of the observed frequencies of the different genotype distribution with their expected frequency under HW in the controls using Pearson’s chi-square (χ^2^) statistical test [24]. Pearson’s chi-square (χ^2^) statistical test was used to compare the alleles difference between the cases and controls. A two-sided *P* value < 0.05 was considered statistically significant.

## Results

There was no significant difference in the age, parity, BMI and the other characteristics between the cases (preeclampsia) and the controls (180 women in each arm), Table 1. There were 63 (35.0%) and 117 (65.0%) women with severe and mild preeclampsia, respectively. The cases of preeclampsia were early preeclampsia in 24 (13.3%) women only and the remaining women 156 (86.7%) had late preeclampsia. Genotypes distribution of *Factor-V Leiden1691G/A* in controls was in accordance with the HWE (*P* > 0.05).

**Table 1:** Comparing the mean (SD) of the sociodemographic characteristics between women with preeclampsia and the controls

Variables	Preeclampsia *N* = (180)	Controls *N* = (180)	*P-value*
Age, year	28.9 (5.2)	28.8 (4.9)	0.884
Parity	3.0 (1.8)	2.7 (1.7)	0.071
Body mass index, Kg/m^2^	24.5 (2.2)	24.5 (2.2)	0.995
Hemoglobin, g/dl	10.3 (1.4)	10.6 (1.6)	0.101

The Factor-V Leiden-variation was present in 9.6% of patients compared to 0.6% of the controls, *P* < 0.001 (OR = 18.60, 95% CI = 2.38–136.1). The homozygous variation A/A were detected Only in 4 patients (6.3%) with severe preeclampsia while, none of the controls, Table 2. Prothrombin G20210A variations were not detected, neither in cases nor in controls.

**Table 2:** Comparing the genotypes and alleles of Factor-V Leiden between women with preeclampsia and the controls

Genotypes	Preeclampsia (180)		Controls (180)		OR (95% CI)	*P-value*
	N	%	N	%		
GG	163	90.6	179	99.4	Reference	
GA	13	7.2	1	0.6	14.2 (1.84–110.3)	0.011
AA	4	2.2	0	0	9.88 (0.52–185.1)	0.053
GA + AA	17	9.6	1	0.6	18.60 (2.38–136.1)	< 0.001
Allele A	21	5.8	1	0.3	22.24 (2.97–166.30)	< 0.001
Allele G	339	94.2	359	99.7	Reference	

*OR* Odds ratio

## Discussion

The main findings of the current study were the significant association between Factor-V Leiden-variation and preeclampsia. We have recently observed that Sudanese women with severe preeclampsia (compared with controls) had significantly higher levels of thrombin-activatable fibrinolysis inhibitor, and significantly lower plasminogen-activated inhibitor 2 level [25]. In line with our findings (association between Factor-V Leiden and preeclampsia) previous studies reported significant associations between the preeclampsia and Factor-V [8–10]. Interestingly in their a meta-analysis Dudding et al [26] showed that Factor-V Leiden increased the risk of preeclampsia by almost 50%. Likewise Lin and August [27] in their systemic review and meta-analyses which included 31 studies (7522 patients) reported that over all women with Factor-V Leiden were at 1.81 higher risk of preeclampsia. In contrary previous study found no association between preeclampsia and factor V Leiden [11, 12]. A systematically review showed no association of Factor-V Leiden with preeclampsia [28].

In the current study Factor-V Leiden-variation was present in 9.6% of women with preeclampsia compared with 0.6% of the controls. A similar rate (9.1%) of Factor-V Leiden was reported in four of 44 preeclamptic women in Tunisia [19]. This rate (9.6%) of Factor V Leiden in our study was lower than rate of the Factor-V Leiden variation reported among patients with preeclampsia in different setting e.g. 15 and 20% in Germany [29, 30], 26% in Israel [31], 15% in Sweden [32] and 18.8% in Hungaria [10]. However, a lower rate in Lindqvist’s prospective study, only 1.9% of pregnant women with Factor-V Leiden variation developed preeclampsia, compared to 1.5% without the variation [32]. Likewise, a lower rate of Factor-V Leiden was reported in preeclamptic women in Italy 7.2–5.2% [8, 33]. In south Africa Factor-V Leiden was not detected in either the preeclamptic women or the control groups [20]. It is worth to be mentioned that our results should be compared with cautious with the results of these studies. Firstly, the difference in ethnicity in the different settings should be remembered. Secondly, types of preeclampsia were enrolled in the different studies e.g. severe and mild, early and late preeclampsia.

We did not detect Prothrombin gene variations neither in the cases nor in the controls. This in line with previous studies reported results [8, 9, 12, 31, 33, 34]. Furthermore, a systematic reviews of only prospective cohort studies [28] showed no associations. Interestingly in South African prothrombin polymorphisms, the variant gene allele was not detected in the investigated cases (preeclampsia) or control groups [20].

## Conclusions

In this study high prevalence of Factor V Leiden gene variation G1691A compared to healthy controls is observed, which provide an evidence of involvement of Factor V Leiden in the precipitations of preeclampsia in Sudanese women. Prothrombin gene variation G20210A is not associated with preeclampsia in this setting.

## Abbreviations

BMI: Body mass index
EDTA: Ethylene diamine tetra acetic acid
HELLP syndrome: Hemolysis, elevated liver enzymes, low platelet count
HWE: Hardy–Weinberg Equilibrium
PCR: Polymerase chain reaction
PCR–RFLP: Polymerase chain reaction–restriction fragment length polymorphism
TBE: Tris Base/Borate/EDTA
UV: Ultra Violet
